# Exploring multitrophic interactions in oilseed rape fields reveals the prevailing role of Carabidae

**DOI:** 10.1002/ece3.8229

**Published:** 2021-10-18

**Authors:** Lola Serée, Antoine Gardarin, Olivier Crouzet, Aude Barbottin, Muriel Valantin‐Morison, François Chiron

**Affiliations:** ^1^ Agronomie INRAE AgroParisTech Université Paris‐Saclay Thiverval‐Grignon France; ^2^ Ecologie Systématique Evolution Université Paris‐Saclay CNRS AgroParisTech Orsay France; ^3^ Office Français de la Biodiversité (OFB) Auffargis France; ^4^ SADAPT INRAE AgroParisTech Université Paris‐Saclay Thiverval‐Grignon France

**Keywords:** avian predation, biological control, food web, functional diversity, trophic‐level interactions

## Abstract

In cropped fields, birds are often at the highest position in the food chain, feeding on pest arthropods and their intermediate predators in a process known as intraguild predation. The net effects of bird predation on phytophagous insect populations (feeding on plants) are difficult to predict without comprehensively describing prey–predator communities and their complex interplay. We sampled bird and arthropod communities in 30 oilseed rape fields in the spring of 2019 and 2020 in France. To assess the top‐down control of arthropods by birds, we used a vertebrate exclusion experiment. Using a taxonomic and functional trait‐based approach, we determined the direct and indirect influences of birds on arthropod predators and phytophagous insect populations in arable crops. We observed a negative relationship between the abundance of Carabidae and phytophagous insects but not with the other predator group suggesting the key role of Carabidae on phytophagous insects in agroecosystem. We found no statistical evidence of intraguild predation from birds toward intermediate predators. Despite the lack of overall effect of predator functional diversity on their prey, we highlighted the negative relationship between the functional complementarity (through functional evenness) of Carabidae and the abundance of phytophagous insects. This result suggests that functional complementarity between Carabidae species could help to reduce phytophagous insect populations. We analyzed the effect of agricultural practices on these multitrophic interactions, showing that pesticide intensity only had detrimental effects on Carabidae abundance, while the frequency of tillage did not affect the studied communities. Complementary indices used to depict communities are helpful to better understand the mechanisms underlying trophic relationships.

## Graphical abstract

Using an experimental study design and combining a taxonomic and a functional approach, we assessed multitrophic relationships in an annual crop (oilseed rape). We found no intraguild predation of birds on ground‐dwelling arthropod predators. The abundance of phytophagous insects in oilseed rape was mainly driven by the abundance, the mean trophic level and the functional evenness of Carabidae.

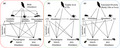

## 1 INTRODUCTION

Recent studies have reported a massive decline in flying insect biomass and bird populations over the last decades (Hallmann et al., 2017; Rosenberg et al., 2019). The intensive use of synthetic pesticides and fertilizers and seminatural habitat loss in agroecosystems are known stressors of biodiversity (Fischer et al., 2018). Birds, particularly those feeding on insect prey during breeding, are highly sensitive to pesticide use (Newton, 2004). The decrease in bird populations has caused important changes to bird community composition. Jeliazkov et al. (2016) stressed the lower trophic level of bird communities in fields sprayed with high doses of pesticides compared to those exposed to low doses. Pesticides are also detrimental to arthropod predators like Carabidae or Araneae species (Geiger et al., 2010; Pekár, 2012). Past and ongoing changes in agroecosystems are thus disrupting the entire trophic network by altering resource provisioning for predators at different trophic levels. The impact of these changes on predator interactions and the cascading effects of vertebrate insectivores on arthropod communities and plants are yet to be fully elucidated.

In cropped fields, the lack of predatory species may disturb the biological control of phytophagous arthropods, which are sometimes perceived as pests (Mäntylä et al., 2011). Theoretical studies on trophic interactions predict that the loss of arthropods and vertebrates feeding on insects will increase herbivore abundance and reduce plant biomass in a process commonly known as trophic cascade (Polis et al., 2000). For instance, in cacao systems, bird and bat exclusion increased phytophagous insect abundance and negatively affected fruit development with a 31% decrease in the final crop yield, despite the concurrent release from predation of intermediate predators such as ants and spiders (Maas et al., 2013). Theoretical predictions suggested that predators of higher trophic levels feeding on intermediate predators, known as intraguild predation, could indirectly weaken the top‐down control of herbivores. Because of their body size and feeding habits, most farmland birds are generalist insectivores during the breeding season, and as top predators, they feed on a diversity of arthropods, including herbivores (e.g., lepidopteran species in *apple orchards*: Mols & Visser, 2002; in *maize*: Maine & Boyles, 2015) and their arthropod predators (King et al., 2015; Orłowski & Karg, 2013). This consumption of intermediate predators such as Araneae, Coccinellidae, or Syrphidae by insectivorous birds might reduce biological pest control (Bosc et al., 2018; Grass et al., 2017; Martin et al., 2013). Communities of intermediate arthropod predators are structured by the abundance of their prey (Warner et al., 2008), whether crop herbivores or alternative prey, as well as by intraguild predation (Roubinet et al., 2017). The overall effect of birds on arthropods is thus difficult to predict without considering the full complexity of food webs. Previous studies of predator–prey interactions often focused on specific predators belonging to one or several arthropod taxa (Mäntylä et al., 2011). In temperate annual crops, however, predator–prey interactions rarely included vertebrates unlike studies conducted in tropical agroforestry systems (Mäntylä et al., 2011).

In addition to the species trophic position, the functional diversity of predators can shape communities of phytophagous insects. Indeed, ecosystem functions depend not on the diversity of species per se but on the diversity of functional characteristics of organisms present in the ecosystem (Hooper et al., 2005). Functional diversity can be defined as the mean value and range of the functional traits of organisms present in a given ecosystem (Díaz & Cabido, 2001). Functional traits are defined as any morphological, physiological, phenological, or behavioral feature measurable at the individual level (Pey et al., 2014). Theory predicts that functionally diverse predatory guilds might partition the prey resource leading to increased herbivore suppression (Frago, 2016). Yet, from a multitrophic‐level approach, higher functional diversity in top predator communities could imply intraguild predation on intermediates predators and thus decrease herbivore suppression. The effects of predator diversity depend on the hunting behavior of predator species and the habitat range of predator and prey species (defined as the spatial extent to which a microhabitat is used by a species (Schmitz, 2007)). Within communities, a higher functional diversity of traits based on diet breadth, habitat domain, and hunting strategy of predaceous arthropods can facilitate biological control (Greenop et al., 2018; Northfield et al., 2017). As a facet of functional diversity, functional evenness between species, defined as the regularity of trait distribution within a community (Mason et al., 2005), may further increase top‐down control. Functional evenness increases resource‐use efficiency in heterogeneous seminatural environments, whether spatially or temporally (Díaz & Cabido, 2001). In perennial crops, diversified microhabitats increase the functional evenness of bird communities with a higher predation rate on plasticine prey (Barbaro et al., 2017). Improving functional diversity and functional evenness in annual temperate crops is crucial to reduce pesticide‐based pest control. Oilseed rape is one of the most cultivated flowering annual crops in France (1 million ha in 2021; Agreste, 2021). However, it is the arable crop receiving the second highest number of insecticide treatments in France (Agreste, 2019). Strategies promoting the biological control of phytophagous insects feeding on this crop are necessary to reduce insecticide use (Lundin et al., 2020). Understanding the relative contribution of functional and taxonomic biodiversity to top‐down control in crops like oilseed rape is thus essential (Gagic et al., 2015).

This study aimed to test the theoretical cascading effects of farmland birds on arthropod communities (i.e., ground‐dwelling predators, phytophagous insects, and alternative prey) in oilseed rape and, especially, the interrelations between ground‐dwelling predators. In this crop, *Brassicogethes spp*. (Coleoptera: *Nitidulidae*), *Psylliodes spp*. (Coleoptera: *Chrysomelidae*), and *Ceutorhynchus spp*. (Coleoptera: *Curculionidea*) are the main pests. These phytophagous insects can be consumed by birds (Gagnon, 2017). At their last instar larvae, these insects fall from the plant to pupate in the soil and are exposed to ground‐dwelling arthropod predators (Riggi et al., 2017), suggesting possible synergies and complementarity in prey consumption between arthropods and birds. In this work, the influence of bird predation on arthropod communities was assessed using an experimental design that excluded birds from sections of the fields. Trophic‐level effects were investigated using a taxonomic (abundance of taxonomic groups) and functional approach. For the latter, we tested whether the single‐trait metrics of functional composition such as mean trophic level (Jeliazkov et al., 2016) perform and functional multitrait metrics. As pesticides and tillage frequency may influence arthropod and vertebrate populations (Chiron et al., 2014; Geiger et al., 2010), the trophic network was analyzed by controlling for the effect of agricultural practices.

We made the following hypotheses: (i) predator numbers (i.e., bird and intermediate ground‐dwelling predator abundance) have an overall direct negative effect on phytophagous insect abundance, or alternatively (ii) a trophic cascade effect from top predators to intermediate ground‐dwelling predator communities can alter the abundance of phytophagous insects. Additionally, we expected (iii) mean trophic level, functional diversity, and functional complementarity of predator communities to have a negative effect on the abundance of phytophagous insects and alternative prey.

## 2 MATERIALS AND METHODS

### 2.1 Study sites and field management surveys

This study was conducted in northern France in an area over 7000 km^2^ (Figure S1). This region is characterized by the intensive cultivation of arable crops (wheat, oilseed rape, and barley) and open‐field landscapes.

In 2019 and 2020, we respectively selected 17 and 13 oilseed rape fields with contrasting cropping systems to cover a gradient of pesticide uses and tillage operations. We determined the cropping techniques of each sampled field by interviewing farmers. We collected detailed information about tillage operations from soil preparation for sowing to harvest (e.g., ploughing, stubble ploughing, hoeing) as well as the herbicides, fungicides, insecticides, and molluscicides sprayed on the field (name, date, doses, and treated area). Based on this information, we calculated two continuous variables: the number of tillage operations carried out during the growing season and the treatment frequency index for all pesticides combined (TFI: number of reference doses applied per hectare; Sattler et al., 2007) (Table S1).

We digitized the land cover types (i.e., cropped and noncropped) within a 500‐m radius around each field based on intensive land surveys from the year of biodiversity sampling using QGIS software (version 2.18; www.qgis.org). Barbaro et al. (2017) showed that bird communities best respond to landscape metrics within a 500‐m buffer. We thus calculated the proportion of seminatural habitats (including woody habitats, fallows, hedges, and wildflowers strips) within each buffer. The proportion of seminatural cover types within a radius of 500 m was between 0 and 28% (median = 4%) of the landscape buffer areas. Since the majority were in open landscapes (only two buffers above 15%) without substantial variations in seminatural cover types, this variable was excluded from our analyses. Moreover, the effect of landscape applied to the cage and control plot located in the same field, thus minimizing landscape bias.

### 2.2 Experimental design

**FIGURE 1:**
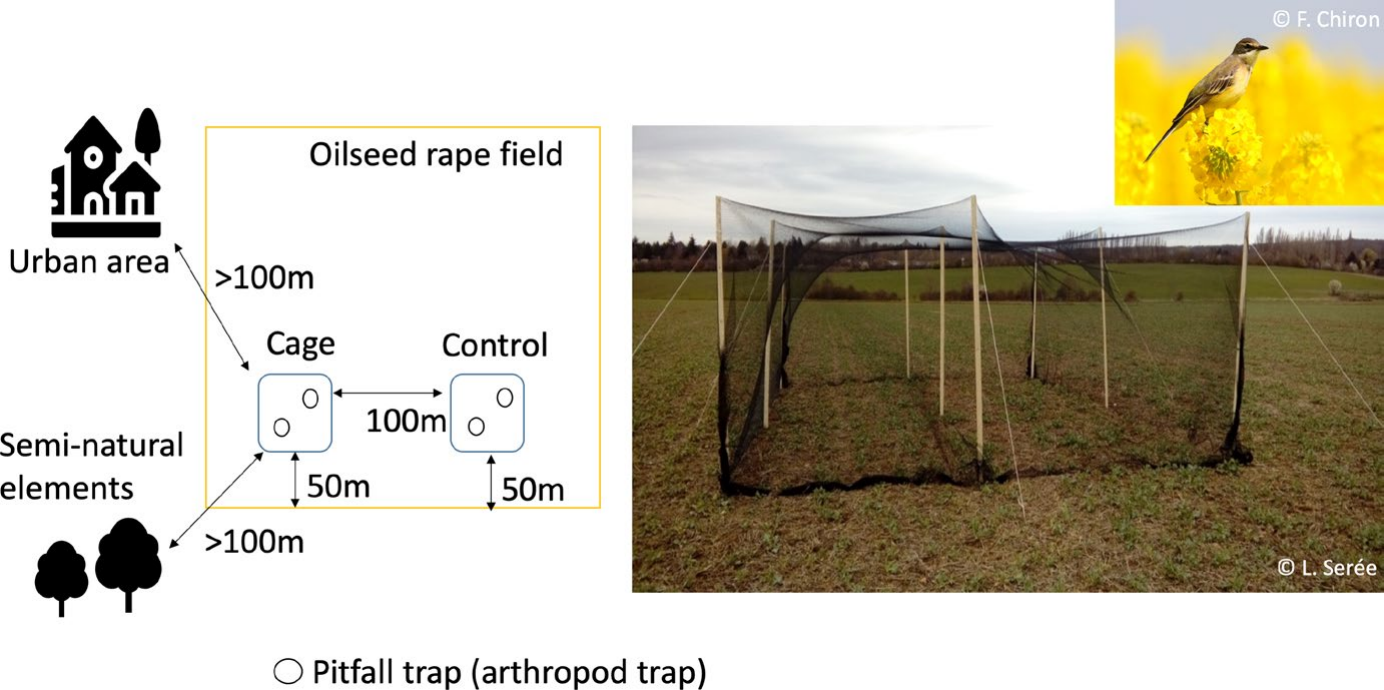
Design of the bird exclusion experiment, photograph of a bird exclusion cage in an oilseed rape field in late February and photograph of a Western Yellow Wagtail (*Motacilla flava*) on oilseed rape

To study the effect of birds on arthropods, we used an exclusion design (Figure 1). Exclusion cages were set up with wooden sticks and measured 4.50 × 6.50 m^2^, with a height of 2.20 m. A net (mesh size = 25.4 mm × 25.4 mm) covered the cage to allow the movement of arthropods but not aerial vertebrates (birds and bats). We expected no effect of bats in our experiment because prey studied at this time are diurnal and within the crop canopy, not flying, thus we did not consider bat populations effect on trophic chain in this study. Exclusion cages were positioned 50 m from the field edge and at least 100 m from a seminatural element or urban area. On each field, we defined a control plot of the same size as the exclusion cage (but without the net) situated 100 m away from the cage and at the same distance from the field edge or other landscape elements. The shortest distance between two sites was 380 m. Oilseed rape fields were sown between 20 August and 10 September. We set up the cages in late February until harvest in early July.

### 2.3 Arthropod sampling

Arthropod sampling occurred mid‐May at the end of flowering stage when most herbivore larvae (*Psylliodes chrysocephala*, *Ceutorrhynchus* spp., and *Brassicogethes aeneus*) that fed within the oilseed rape petioles, stems, and flowers leave the plant to pupate in the soil. These larvae become highly exposed to predation by ground‐dwelling arthropods and birds (Gagnon, 2017; Riggi et al., 2017).

Two pitfall traps filled with 150 ml of saltwater solution (50 g/L salt, with 2 ml/L of odorless detergent) were used to catch arthropods for 7 days in each cage and control plot. We identified Carabidae and Araneae at the species level. We also distinguished Chilopoda (class), Opiliones (order), Staphylinidae (family), Collembola (class), Acari (subclass), Thysanoptera (order), Cicadomorpha (infra‐order), Curculionidae (family), Nitidulidae (family), and Chrysomelidae (family). We pooled the activity densities (hereafter, abundances for simplicity) of arthropods in the two pitfall traps for each treatment (i.e., cage and control plot). We summed the abundances of Chilopoda, Opiliones, and Staphylinidae in the “other predator” group and Collembola and Acari in the “alternative prey” group. Thysanoptera, Cicadomorpha, Curculionidae, Nitidulidae, and Chrysomelidae were grouped as “phytophagous insects” (see Table S1 for a summary of data).

### 2.4 Bird surveys

Each year during spring, two birders visited the control plot twice early in the morning (6:00–10:30 am) in the absence of wind, rain, and fog. The first visit occurred from 8 to 23 April (to detect early‐season breeders) and the second from 16 to 29 May (late‐season breeders). As the detectability of birds is influenced by observer experience, count duration, and distance to the observer, skilled birders identified all bird species within a 100‐m radius around the control plot and during 10 min each visit. We rotated the order of the points surveyed between two visits to minimize time‐of‐day effects. Each plot was always surveyed by the same birder. The two visits allowed us to identify the full breeding bird community during the insect sampling period. For each species, the sum of abundances for the two visits was used as a standardized estimate of abundance per plot for further analyses. Species richness was the total number of species.

### 2.5 Bird, Araneae, and Carabidae functional traits

To compute the functional multitrait indices of predator communities, we selected relevant traits as key indicators of species effect or response to predation (Philpott et al., 2009; Rusch et al., 2015). For birds, we built a species‐trait matrix based on the breeding period, hunting strategy, stratum use, body size, habitat specialization, proportion of vertebrates, arthropods, and plants in the diet, and mean number of eggs laid per year (Chiron et al., 2014; Julliard et al., 2006; Storchová & Hořák, 2018). For Carabidae and Araneae, we retained the daily and annual activity periods, dispersal mode, stratum use, hunting strategy, and body size. For Carabidae, we added the proportions of plants, detritivore, and living prey in the diet (Tables S2 and S3 for hypotheses underlying the choice of traits and their sources).

To consider diet variations within predatory communities, we computed the community trophic index (CTI) as a proxy of “trophic height” (Jeliazkov et al., 2016). Since Araneae predate arthropods, we did not calculate their CTI. CTI values ranged from 1 (all species of the sampled community are at the “lower level” of the trophic chain with birds eating plants and Carabidae eating detritus) to 3 (all species are carnivorous) (see Appendix S1 for more details).

### 2.6 Functional community indices

To assess the effect of predator functional diversity (i.e., birds, Carabidae, and Araneae) on the food web, we calculated three trait‐based indices: functional evenness (Feve), functional diversity via Rao's quadratic entropy (RaoQ), and functional dissimilarity (Fdis) using the FD package (Laliberté et al., 2016). Feve describes the regularity of abundance distribution in a functional trait space (Villéger et al., 2008), while RaoQ describes the variation of species trait composition within the community, weighted by their relative abundance (Spake et al., 2016). Fdis is the mean distance of individual species to the centroid of all species in the multidimensional trait space (é & Legendre, 2010). Due to its collinearity with the RaoQ index (*r* cor = .96; Figure S2), we report Fdis results in Appendix S4. Because species traits were represented by a combination of continuous and categorical variables, we used Gower's method to calculate the distance matrix and then standardized all trait scores (Laliberté & Legendre, 2010).

### 2.7 Data analysis

All analyses were carried out using R version 3.5.2 (2018‐12‐20). We divided our analyses into two steps. First, we analyzed variations in abundance, CTI, and functional diversity indices per taxonomical group (birds, Carabidae, and Araneae) between the cages and control plots (experimental treatments). We ran generalized linear mixed models fitted with penalized quasi‐likelihood and assuming Poisson (for abundance) or Gaussian distributions of errors (for CTI and functional diversity indices) using glmmPQL (MASS package). We included “year,” experimental treatment, and their interaction as fixed effects. To consider the nested design and spatial dependence between control and cage plots, we used “field name” as a random component. We assessed the statistical differences between plots using ANOVA of type II and post hoc tests (lsmeans package). Complete results are presented in Table S4.

**FIGURE 2:**
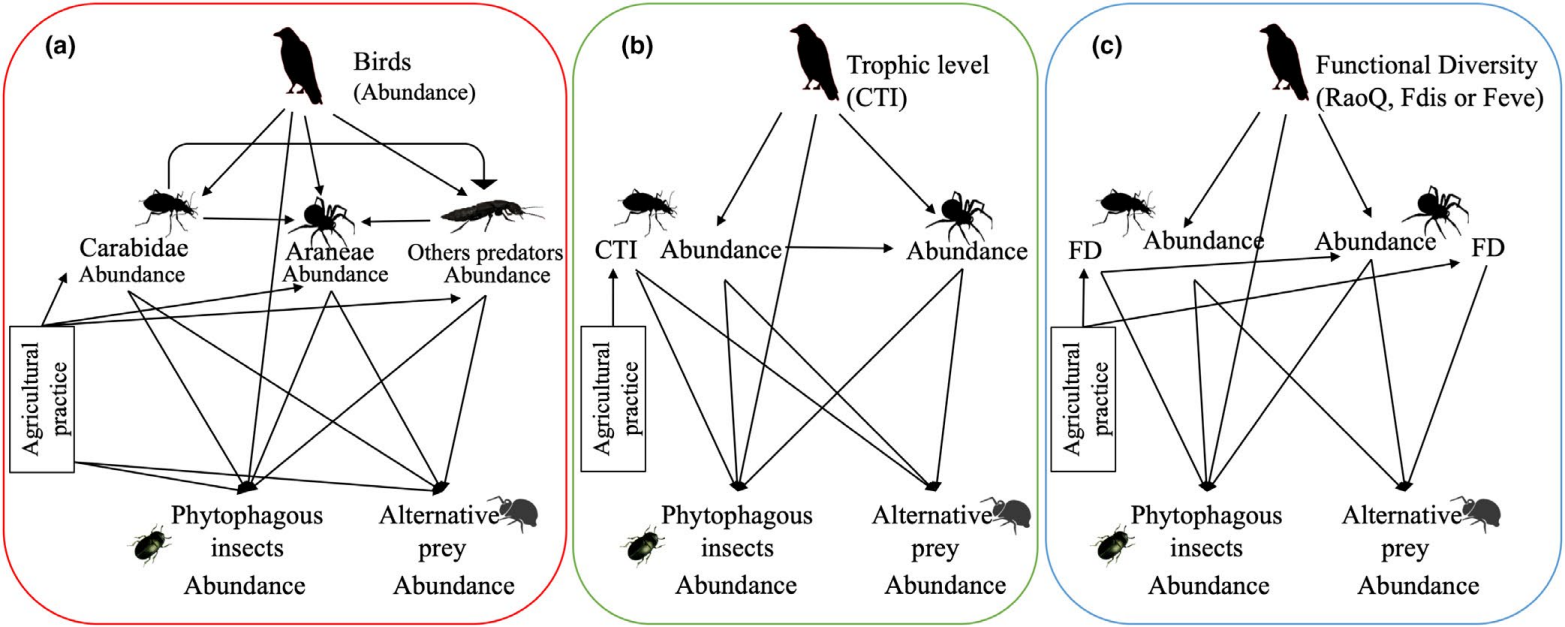
Hypothetical food web relationship using structural equation models in oilseed rape crop. (a) taxonomic approach based on abundance; (b) trophic approach with community trophic index (CTI); (c) functional approach based on functional diversity (FD) indices with Rao's quadratic entropy (RaoQ), functional evenness (Feve), or functional dissimilarities (Fdis) depending on the model

To assess trophic cascade effects, we evaluated multitrophic prey–predator relationships with structural equation modeling (SEM) using the “piecewiseSEM” R package (Lefcheck, 2016). This method is based on path analyses, where each path represents a hypothesized causal relationship. We took birds as top predators of phytophagous insects, intermediate ground‐dwelling predators, or alternative prey. We also tested predation from Carabidae and other predators to Araneae and the link between Carabidae and other predators. Since year had no substantial effect on arthropod and bird abundance data, we merged the data from the two annual campaigns into a single dataset. We included all collected abundance data (cage and control) by assigning 0 to bird abundance in the cage treatment. We computed the marginal *R* ^2^ (considering only the fixed effect variance) and the estimates for each relationship. Following Grace et al. (2018), we calculated standardized estimates (std.estimates) based on the observational‐empirical approach using *R* ^2^, because it could not be calculated for glmmPQL class models (Appendix S2). We tested 10 models separately: models 1 and 2 described the relationships between taxa based on their abundances (approach A), models 3 and 4 based on CTI indices (approach B), and models 5–10 based on the three aforementioned functional indices of predatory groups (RaoQ, Feve, and Fdis; approach C). To avoid model overparametrization, we independently tested models with only pesticide (TFI, models 1, 3, 5 and 7) or tillage frequencies (models 2, 4, 6, and 8). We removed “other predators” from CTI and functional diversity‐based models, because *Chilopoda*, *Opiliones*, and *Staphylinidae* could not be identified at the species level (Figure 2).

Because of overdispersion with glmer (both with “Poisson” or “Negative binomial” distribution), we used glmmPQL mixed models (using penalized quasi‐likelihood and Wald F tests; Bolker et al., 2009; Breslow & Clayton, 1993; Venables & Ripley, 2002), assuming a “Poisson” (for count data like abundances) or “Gaussian” distribution (for functional indices), with “field name” as the random component. We assessed the overall fit of the piecewise SEM using Shipley's test of direct separation (*χ* ^2^ of Fisher's C test statistics) and checked whether the models could be improved with the inclusion of any of the missing paths. For abundance‐based models (approach A), we added a link between alternative prey and phytophagous insects. We rejected models if *χ* ^2^ fell below the significance level (*p *< .05), indicating the inconsistency of the model with the data. For each approach (A, B, C), we summarized the statistical results for all tested models (Table 1) and graphically depicted the SEM network for models within a ΔAIC < 2 from the best‐supported model.

**TABLE 1:** Statistical results of the models assessing the multitrophic network

Approach	Models	Agricultural practice variable	Fischer's *c* test	df	*p*‐Value	ΔAIC
A (Abundances)	1	TFI	5.11	2	.08	1.12
	2	Tillage	4.99	2	.08	0.00
B (Mean trophic level)	3	TFI	28.90	20	.09	3.89
	4	Tillage	25.08	20	.20	0.00
C Functional diversity (RaoQ)	5	TFI	36.84	26	.08	3.94
	6	Tillage	32.90	26	.17	0.00
C Functional diversity (Feve)	7	TFI	35.16	26	.11	3.19
	8	Tillage	31.97	26	.19	0.00

Note

We modeled networks using a taxonomic approach A (abundance of taxa), a trophic‐level approach B (CTI), and a trait‐based functional approach C (RaoQ and Feve). To avoid model overparametrization, the model structure alternatively included the treatment frequency index (TFI) or tillage frequency (Tillage) as agricultural practices. Delta AIC from the best model is given.

The effect of agricultural practices (i.e., TFI and tillage frequency) and the proportion of seminatural habitat within a 500‐m radius could not be directly related to bird abundance due to the sampling design with exclusion cages. We thus tested the effect of agricultural practices and landscape composition on the abundance of birds on a separate model on control plots only. We used a mixed model (glmmPQL) assuming a “Poisson” distribution on data collected on control plots to check for confounding effects on trophic interactions. Spatial correlation in the residuals was checked with Correlogram and the “spline.correlog” function from the “ncf” package. No spatial correlation was detected. Pesticide application (value=−0.06; df = 13; *t*‐value = −0.57; *p* = .58), tillage frequency (value = −0.16; df = 13; *t*‐value = −1.62; *p* = .13), and the proportion of semi‐natural habitat (value = −0.01; df = 13; *t*‐value = −0.07; *p *= .95) had no effect on bird abundance.

## 3 RESULTS

Alternative prey (mean = 845.0; min–max = 64–3729) represented the highest number of trapped individuals per taxonomic group in the 30 fields sampled in 2019 and 2020, including both control and cage plots. Few phytophagous insects were recorded (mean = 8.7; min–max = 0–39). The abundance of ground‐dwelling predators ranged from 11 to 114 individuals (mean = 71.3), with a similar abundance of Carabidae (mean = 29.0; min–max = 5–85) and Araneae (mean = 28.5; min–max = 0–92), while the other predator group was half as abundant (mean = 13.7; min–max = 1–55). The total number of birds n control plots ranged from 2 to 25 individuals (mean = 11.7). The three most abundant bird species were *Alauda arvensis* (Eurasian skylark) (26% of total bird abundance), *Sylvia communis* (Common whitethroat) (19%), and *Emberiza calandra* (Corn bunting) (11%) (see Figure S3 for the full species list).

### 3.1 Bird exclusion effects on arthropods

**FIGURE 3:**
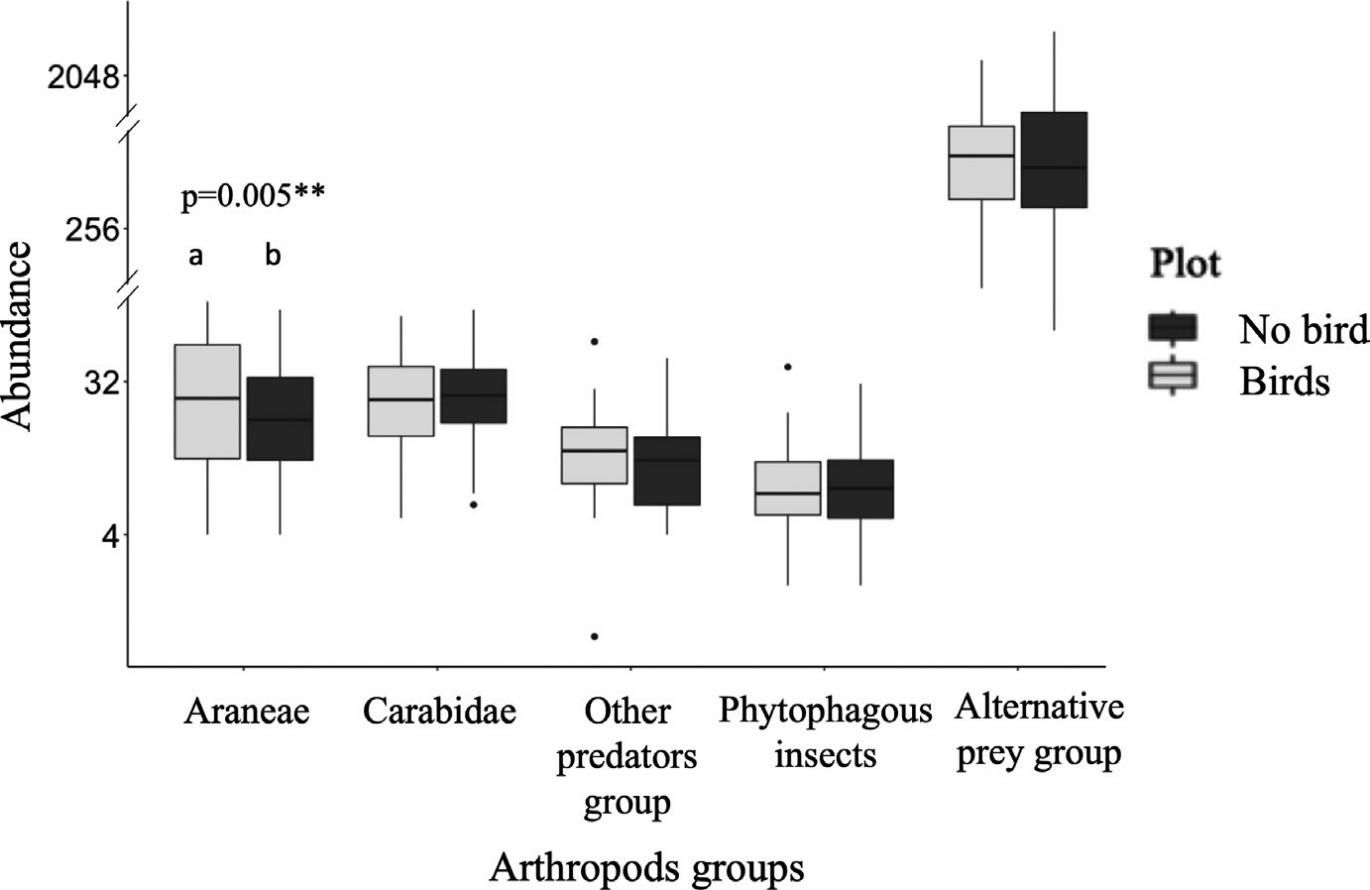
Boxplots of the number of arthropods with and without birds. “Other predators” corresponds to the sum of Staphylinidae, Opiliones, and Chilopoda abundances. “All phytophagous insects” include Thysanoptera, Cicadomorpha, Curculionidae, Nitidulidae, and Chrysomelidae. “Alternative prey” includes Collembola and Acari

Araneae were less abundant in the bird exclusion plot (−24.4%) than the control plot (Chisq = 8.02; *p* = .005; Figure 3; Table S4 for complete results). We observed no significant effect of bird exclusion on the abundance of Carabidae, other predators, phytophagous insects, or alternative prey (Table S4).

### 3.2 Effects of bird abundance on arthropods (approach A)

For the abundance‐based models, models 1 and 2 fitted the data weakly (Fisher's *C* = 5.113, df = 2, *p* = .078 for model 1 and Fisher's *C* = 4.993, df = 2, *p *= .082 for model 2; Tables 1 and S5).

**FIGURE 4:**
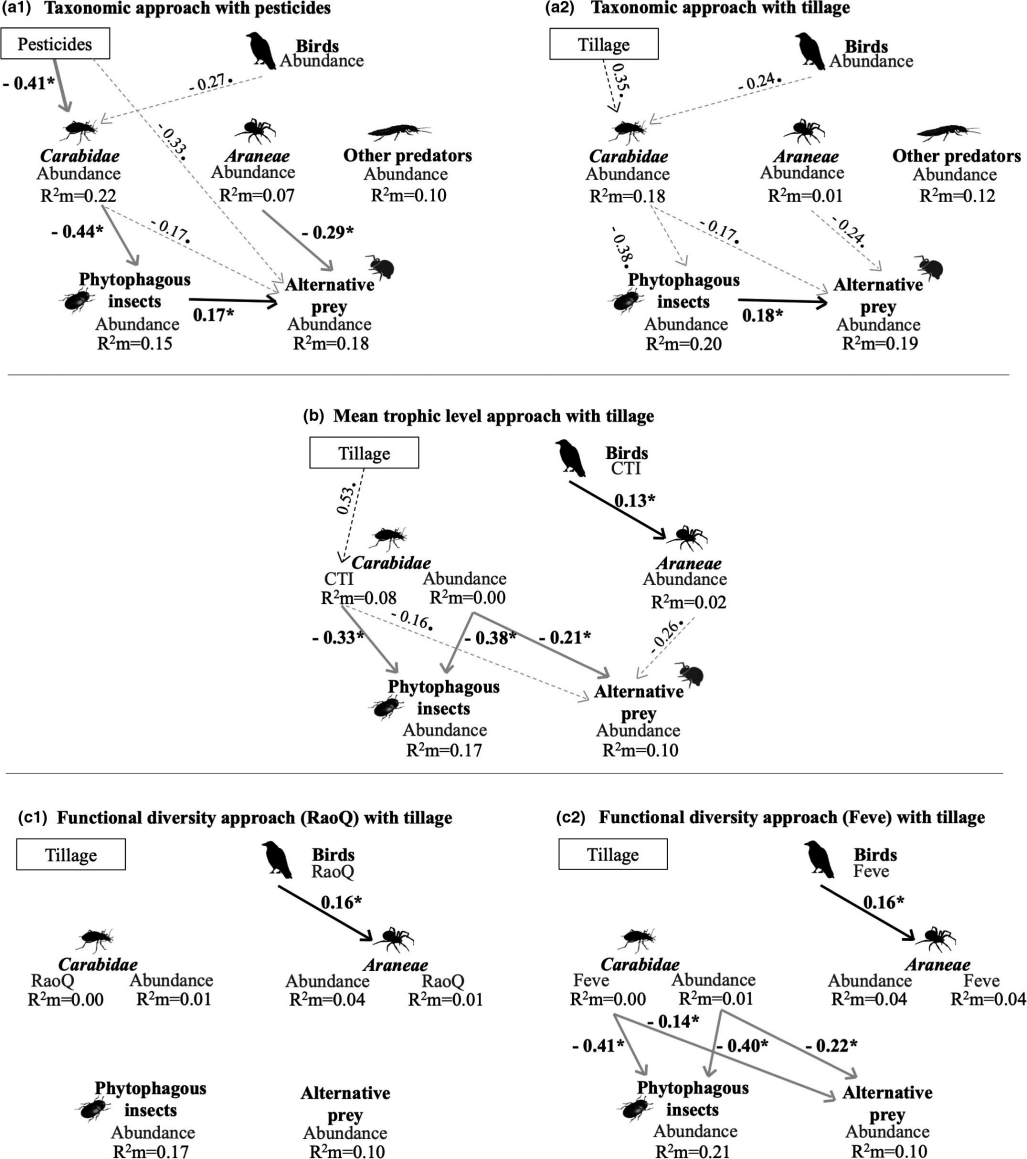
Structural equation modeling showing direct and indirect relationships between (a) the abundance of predators and phytophagous arthropods (model 1 in a‐1 and 2 in a‐2; Table 1), (b) the mean trophic level of predators (model 4; Table 1), and (c) the functional diversity (RaoQ) and functional evenness (Feve) of predators on the one hand and the abundance of phytophagous arthropods on the other (model 6 in c‐1 and 8 in c‐2; Table 1). Black and grey lines indicate positive and negative relationships, respectively. Solid lines represent significant relationships (*p *< .05) and dotted lines represent trends (0.05 < *p *< .10). We reported standardized estimates (with “*” for significant relationships and “.” for trends) for each arrow and marginal *R* ^2^ (*R* ^2^ *m*). Other predators correspond to the sum of abundances of Staphylinidae, Opiliones, and Chilopoda individuals. Phytophagous insects include Thysanoptera, Cicadomorpha, Curculionidae, Nitidulidae, and Chrysomelidae. Pesticide is the treatment frequency index

In model 1 with pesticide use (TFI), phytophagous insect abundance was negatively related to Carabidae abundance (std.estimate = −0.44; *p *= .048; Figure 4a1; Table S6). Alternative prey was negatively related to Araneae (std.estimate = −0.29; *p* = .027; Figure 4a1; Table S5), and a negative trend was observed with Carabidae (std.estimate = −0.17; *p* = .063; Figure 4a1; Table S5). The abundances of Carabidae, other predators, and Araneae were unrelated. Birds were not significantly related to any other taxa, although a negative trend was detected between bird and Carabidae abundances (std.estimate = −0.27; *p* = .058; Table S5). Carabidae abundance was negatively related to pesticide use (TFI) (std.estimate = −0.41; *p* = .048; Table S5), while phytophagous insects and other taxonomical groups were not (Figure 4). These relationships were no longer significant in model 2 with tillage frequency (Figure 4a2; Table S6).

### 3.3 Effects of predator trophic level on prey abundance (approach B)

Bird communities were composed of seed‐ and insectivorous‐eating species, with very few vertebrate predators (mean CTI = 1.46, min–max = 1.2–1.8; Table S1), while Carabidae communities were mainly carnivorous (mean CTI = 2.57, min–max = 2.31–2.79).

Carabidae and Araneae were poorly explained in these models (low marginal *R* ^2^). However, Araneae abundance was slightly positively related to the trophic level of the bird community (std.estimate = 0.13; *p* = .01; Figure 4b; Table S7). In addition to the abovementioned negative relation with Carabidae abundance, phytophagous insects were also negatively related to the trophic level of Carabidae communities (std.estimate = −0.33; *p* = .02; Figure 4b; Table S7).

### 3.4 Effects of predator functional diversity on prey abundance (approach C)

We found weak but positive and significant relationships between Araneae abundance and the functional diversity (RaoQ) or evenness (Feve) of birds (Figure 4). No relationship was observed between phytophagous insect or alternative prey abundance and the functional diversity (RaoQ) of their predators represented by Carabidae, Araneae, and birds (Figure 4c1; model 10 in Table S8). Phytophagous insect and alternative prey abundance was nevertheless negatively related to Carabidae functional evenness (Feve) and abundance (Figure 4c2; models 6 and 8 in Table S8).

## 4 DISCUSSION

Our study highlights that the abundance of phytophagous insects in oilseed rape was mainly driven by the abundance, mean trophic level, and functional evenness of Carabidae communities. Bird and other predator abundances were not connected to other groups, while Araneae abundance was only negatively related to the abundance of alternative prey. Thus, birds did not contribute to or disrupt the other putative trophic relationships.

### 4.1 Carabidae as the only ground‐dwelling predator negatively related to phytophagous insect communities

Only Carabidae abundance was negatively related to phytophagous insect abundance, while both Carabidae and Araneae were negatively related to alternative prey, suggesting a top‐down control effect. Since the negative relationship between phytophagous insects and Carabidae was stronger than with alternative prey, this is unlikely to reduce the impact of generalist predators on pest populations. Nevertheless, it may help them survive periods of low pest availability (Leroux & Loreau, 2008). The unexpected positive relationship between phytophagous insect and alternative prey abundances could relate to the favorable soil conditions for both prey groups. For instance, pollen beetle and epigeal Collembola have a higher mortality when the proportion of soil clay is high (Filho et al., 2016; Riggi et al., 2017). We did not detect any relationship between birds, Araneae, and other ground‐dwelling predators with phytophagous insects. This could be due to the low recorded abundance of phytophagous insects or to a mismatch between the spatial niche of birds and Araneae toward phytophagous insects collected on the soil surface in spring. When oilseed rape is at a reproductive stage, birds might prospect in tall dense vegetation rather than on the soil surface. This might have a limited effect on the abundance of ground‐dwelling predators and phytophagous arthropods such as stem flea beetles, pollen beetles, and weevils, which are mainly exposed to predation on the soil surface when they fall to pupate in the soil in spring. Birds are probably more able to feed on foliar or flying insects. For instance, Sylviidae species (e.g., the common whitethroat *Sylvia communis*, the second most abundant species in our study) can feed on aphids at the top of the oilseed rape plant, while *Alauda arvensis*, skylark, needs more open vegetation to feed on the ground. Yet the existence of open or heterogeneous crop covers depends on the sowing conditions, farming practices, and cropping systems. Further fieldwork and analysis at different crop growth stages coupling behavioral monitoring and sentinel prey experiments are needed to describe prey–predator relationships.

In addition to abundance, the mean trophic level of Carabidae was negatively related to the abundance of phytophagous insects, indicating that the higher proportion of predatory species in Carabidae communities promotes phytophagous regulation. The diet of species in the predator community was therefore relevant for Carabidae. Nevertheless, accounting for the proportion of predatory bird species did not reveal any relationship between birds and arthropods, perhaps because of the relatively low number of insectivorous birds in these homogeneous and simplified agricultural landscapes (Jeliazkov et al., 2016).

### 4.2 Intraguild predation not disrupting links between ground‐dwelling arthropods and their prey

Although birds likely predate on other natural enemies like ground‐dwelling predators including spiders and carabids species, we found no evidence that they disrupt biological control exerted by these arthropods contrary to our second hypothesis. Indeed, the absence of relationships suggests that birds do not disrupt the biological control exerted by Carabidae. As suggested by Martin et al. (2013) who studied lepidopteran pest on cabbage, birds in open agricultural landscapes mostly have a pest reduction effect rather than an antagonistic effect on biological control. Nevertheless, we cannot exclude intraguild predation from birds to foliar natural enemies as it was not investigated in this study. Moreover, as birds switch their prey and behavior during the breeding season in response to the needs of their young (Grass et al., 2017; Naef‐Daenzer et al., 2000), it could be interesting to investigate multitrophic relationships at different times throughout the year. For instance, later in the summer, birds like swallows (Hirundinidae) and swifts (Apodidae) feed on the new adult generation of pollen beetle or weevils (Orłowski et al., 2014).

The unexpected increase in Araneae abundance when birds were present (i.e., control plot versus cage) suggested no intraguild predation conversely to previous studies (Bosc et al., 2018; Gras et al., 2016; Maas et al., 2013; Mestre et al., 2013). However, our results provided no reliable explanations for this positive relationship, which did not hold true in SEM models based on abundances. We reject a potential bias in the experimental design caused by less Araneae ballooning within the cage due to net filtering, since the proportion of individuals was quite similar in both treatments (98% in the cage vs 97% in the control plot).

### 4.3 Low effect of functional diversity on trophic network in oilseed rape fields

Our results showed a negative relationship between the functional evenness of Carabidae toward phytophagous insects and alternative prey populations. Carabidae communities with a more even distribution of species across the trait space more efficiently reduce prey populations, which supports the functional complementarity hypothesis (Gagic et al., 2015; Sánchez‐Hernández et al., 2021). In the case of predation traits, niche partitioning among Carabidae species enables predation on different prey life stages at different locations or periods during the season, which generally improves biological control (Rusch et al., 2017).

The lack of effect of the functional diversity indices of birds and Araneae could be explained by simplified predator communities due to important biotic and abiotic filters in arable cropping systems. This reduces species diversity and selects species adapted to human‐disturbed environments, leading to communities with a narrow trait range and low functional diversity (Michalko & Birkhofer, 2021). As shown by Barbaro et al. (2017), bird insectivory increases with the functional evenness of avian communities but only in more heterogeneous landscapes. Despite the relatively high amount of insect biomass in oilseed rape compared to other crops in spring, the simplified field landscapes where our experiment was performed did not support functionally diversified bird communities.

### 4.4 Pesticide intensity and tillage effects on abundance, trophic level, and functional diversity indices

Despite the known negative effect of soil disturbance on soil arthropods (Henneron et al., 2015), their abundances were not related to tillage frequency. Arthropod sampling was conducted more than 6 months after the last tillage event and crop sowing. This time interval could have allowed arthropod communities to homogenize over the crop mosaics, thereby preventing us from detecting effects of local soil management.

We found a negative relationship between pesticide use (through TFI) and Carabidae abundances but not with the abundance of other ground‐dwelling predators, neither with those of phytophagous insects and alternative prey. Among pesticides, insecticides have direct lethal or sublethal effects on Carabidae (Holland & Luff, 2000). For instance, in our study, pyrethroids were the main insecticides applied in autumn and spring, reducing the activity of Carabidae and thus decreasing trap captures (Tooming et al., 2014). No effect of pesticides and tillage was found for spiders, perhaps because most of species found in arable crops colonize the crops in early spring after hibernation in surrounding noncrop habitats (Michalko & Birkhofer, 2021), making them lowly sensitive to local management intensity during this period.

## 5 CONCLUSION

Only Carabidae were related to phytophagous insects, while birds, Araneae, and other predators were not, suggesting the prevailing efficiency of Carabidae communities in regulating ground‐dwelling phytophagous insects in agroecosystems.

We showed a negative relationship between the functional evenness of Carabidae (but not functional diversity) and the abundance of their prey. This is supported by previous studies showing the positive effect of the functional evenness of Carabidae abundance on biological control (Crowder et al., 2010). Therefore, cropping systems sustaining community evenness are more likely to promote the regulation of phytophagous insects in annual crops like oilseed rape. Reducing or eliminating pesticide use may also increase Carabidae abundance with expected beneficial effects on biological control. By comparing trophic networks under different metrics, we showed that different facets of Carabidae communities explain trophic relationships, not only their abundance but also the proportion of predatory species and functional evenness, thus leading to a better understanding of biological control mechanisms.

## CONFLICT OF INTEREST

We declare no competing financial interests or personal relationships that could have appeared to influence the work reported in this paper.

## AUTHOR CONTRIBUTIONS

**Lola Serée:** Conceptualization (equal); Formal analysis (lead); Investigation (equal); Methodology (equal); Visualization (equal); Writing‐original draft (lead); Writing‐review & editing (equal). **Antoine Gardarin:** Conceptualization (equal); Methodology (equal); Supervision (equal); Writing‐review & editing (equal). **Olivier Crouzet:** Investigation (equal); Writing‐review & editing (equal). **Aude Barbottin:** Investigation (equal); Writing‐review & editing (equal). **Muriel Valantin‐Morison:** Conceptualization (equal); Supervision (equal); Writing‐review & editing (equal). **François Chiron:** Conceptualization (equal); Investigation (equal); Methodology (equal); Supervision (lead); Writing‐original draft (equal); Writing‐review & editing (equal).

## Supporting information

Supplementary Material

Data S1

## Data Availability

Data are archived on Dryad https://doi.org/10.5061/dryad.0p2ngf22m.
