# Supplementary material for: Exploring multitrophic interactions in oilseed rape fields reveals the prevailing role of Carabidae
Source: Ecol Evol. 2021 Oct 18;11(21):15377–88. doi: 10.1002/ece3.8229 (PMC8571632; doi:10.1002/ece3.8229)
Supplement: Supplementary file 1 — Supplementary Material [file ECE3-11-15377-s001.pdf]

Exploring multitrophic interactions in oilseed rape fields reveals the prevailing role of Carabidae

Lola Serée[1-3], Antoine Gardarin[1], Olivier Crouzet[2], Aude Barbottin[4], Muriel Valantin-Morison[1] & François Chiron[3]

[1] INRAE, AgroParisTech, Université Paris-Saclay, Agronomie, 78850 Thiverval-Grignon, France

[2] Office Français de la Biodiversité (OFB), 78610 Auffargis, France

[3] Université Paris-Saclay, CNRS, AgroParisTech, Ecologie Systématique Evolution, 91405, Orsay, France.

[4] INRAE, AgroParisTech, Université Paris-Saclay, SADAPT, 78850 Thiverval-Grignon, France

Corresponding author: Lola Serée, lola.seree@inrae.fr

Postal address: UMR Agronomie INRAE-AgroParisTech, Avenue Lucien Brétignières, 78 850 Thiverval-Grignon, France

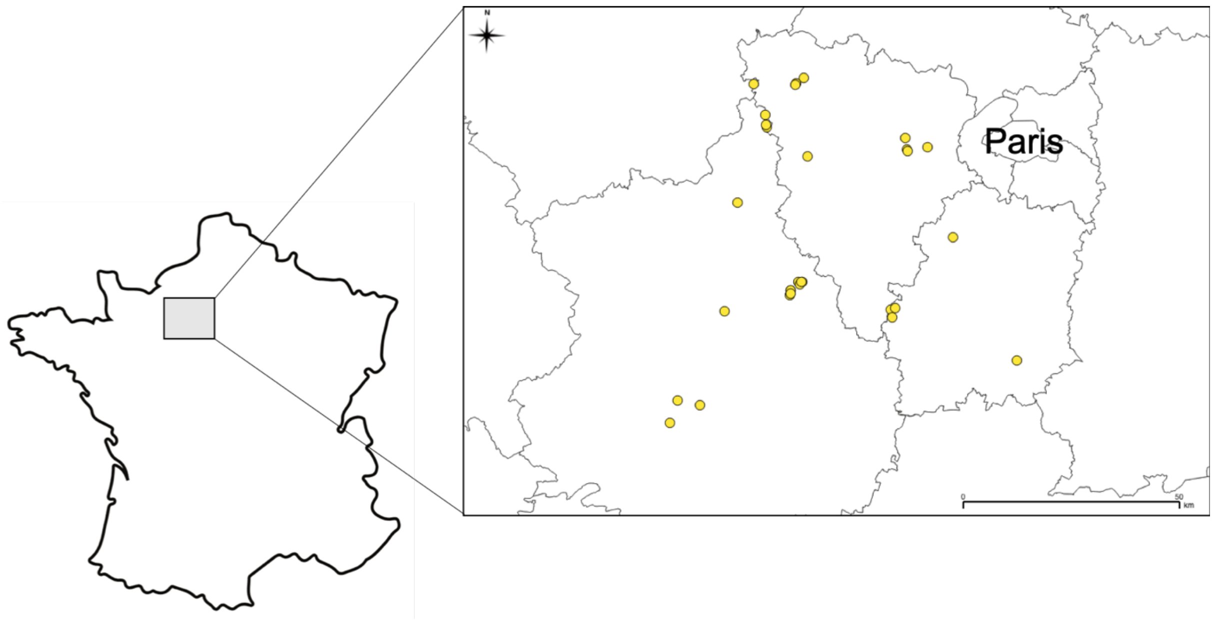

Figure S1: Map of the study sites (yellow points) sampled in 2019 and 2020 in northern France.

Table S1: Summary of data collected (species abundances and richness), mean trophic level (CTI), functional indices, farming practices and landscape context variables across our sampling sites. TFI (Treatment Frequency Index) is the number of reference dose applied per hectare. Tillage number is the sum of the tillage operations from preparing the soil for sowing to harvest (e.g., ploughing, mechanical weeding).

	Cage				Control			
Variables	Mean	SD	Min	Max	Mean	SD	Min	Max
Birds abundance	0	0	0	0	11.7	6.14	2	25
Carabidae abundance	29.67	17.68	6	85	28.37	17.83	5	78
Araneae abundance	25.43	19.96	3	81	31.63	26.44	0	92
Others predator's abundance	13.20	9.05	4	44	14.27	10.28	1	55
Phytophagous abundance	8.60	6.69	0	31	8.97	7.25	2	39
Alternative preys abundance	876.67	794.1	64	3729	813.27	627.5	114	2527
Birds richness	0	0	0	0	4.77	2.03	1	9
Carabidae richness	6.67	1.94	4	12	6.2	2.25	2	11
Araneae richness	6.50	3.41	2	15	8.27	4.64	0	18
Birds Feve	0	0	0	0	0.82	0.11	0.47	0.99
Birds RaoQ	0	0	0	0	0.03	0.01	0	0.06
Carabidae Feve	0.66	0.11	0.34	0.85	0.67	0.16	0.21	0.95
Carabidae RaoQ	0.04	0.02	0.02	0.09	0.04	0.02	0.01	0.07
Araneae Feve	0.72	0.14	0.42	0.99	0.73	0.17	0	0.96
Araneae RaoQ	0.04	0.02	0.01	0.07	0.04	0.02	0	0.06
Bird's CTI	0	0	0	0	1.46	0.12	1.21	1.78
Carabidae CTI	2.58	0.11	2.39	2.79	2.56	0.10	2.31	2.75
TFI total	3.73	1.88	0	6.89	3.73	1.88	0	6.89
Number of tillage	2.03	1.67	0	5	2.03	1.67	0	5
% (0-1) seminatural habitats (500 m)	0.06	0.07	0.001	0.28	0.06	0.07	0.001	0.28
% (0-1) oilseed rape (500 m)	0.18	0.11	0.00	0.38	0.18	0.11	0.00	0.38
Field area (ha)	12.06	7.28	2.95	37.15	12.06	7.28	2.95	37.15

Table S2: Hypotheses underlying the choice of predator functional traits.

Effect trait	Hypothesis
Body size	Body size is an important factor determining predator-prey trophic interactions and the functional response of predators (Brose, 2010).
Daily activity period	The diel rhythm of predators influences the type of prey they encounter (Petersen & Woltz, 2015) and their possible complementarity (e.g. resource partitioning) or antagonism (e.g. intraguild predation).
Annual activity period	The annual rhythm of predators influences the type of prey they encounter and their possible complementarity (e.g. resource partitioning) or antagonism (e.g. intraguild predation) (Hugues, 2009).
Dispersal mode or ability	The dispersal ability is related to the foraging ability and to the attack rate of preys (Forsythe, 2009).
Proportion of animals or plants in the diet	The diet affects the role of generalist predators as biocontrol agents (Roubinet et al., 2017).<br>A higher proportion of invertebrates in diet contribute to a better control of arthropods populations in studied fields (Loeuille et al., 2013).
Stratum use	The preference for low, medium or high vegetation layer determines the putative preys of the predators and their possible complementarity (Northfield et al., 2017; Perović et al., 2018).
Hunting strategy	Together with stratum use, the hunting strategy is one of the main traits determining predator complementarity as it affects the spatial extent of predator movements (Schmitz, 2009).
Habitat specialism (birds only)	The degree of specialization to the habitat is the breadth of species requirement for the habitat and the resources in its largest sense. It defined how specialized or restricted a species is to a given habitat type *versus* habitat use breadth that a more generalist species encompasses. Having a range of species that respond differently to habitat conditions can stabilize ecosystem processes (Clavel et al., 2011).
Breeding activity period (birds only)	The breeding activity period of birds in the season influences the type of prey they encounter (Naef-Daenzer et al., 2000) and their possible complementarity (e.g. resource partitioning) across the entire breeding activity period from March to July.
Number of eggs per year (birds only)	Lack (1954) demonstrated that diet is correlated with clutch size, with herbivores having smaller clutches than other trophic groups. We also hypothesized that more young birds to feed in a single or multiple brood for a given species could lead to a higher pressure on arthropods consumed.

Table S3: Birds, spiders and carabids traits computed in species trait matrix and used in the study

Taxonomic group	Trait	Range or categories	Source
Carabidae	Daily activity period	Diurnal ; nocturnal (1/0)	Cole et al., 2002
	Annual activity period	Autumn ; spring (1/0)	Birkhofer et al., 2015
	Dispersal mode	Flight ; ground	Martin et al., 2019
	Wings morphology (dispersal ability)	Brachypterous ; macropterous ; dimorphic (when both are repertoried)	BETSI Database (Pey et al., 2014) (https://portail.betsi.cnrs.fr/)
	Stratum use	Ground ; vegetation (1/0)	Birkhofer et al., 2015; Martin et al., 2019
	Visual hunter (hunting strategy)	1 = Yes ; 0 = No	Baulechner et al., 2019
	% of zoophagous diet (0-100)	0 - 100	BETSI Database (Pey et al., 2014) (https://portail.betsi.cnrs.fr/)
	% of phytophagous diet (0-100)	0 - 100	BETSI Database (Pey et al., 2014) (https://portail.betsi.cnrs.fr/)
	% of detritivorous diet (0-100)	0 - 5.41	BETSI Database (Pey et al., 2014) (https://portail.betsi.cnrs.fr/)
	Body size (mm)	2.95 – 15.00	BETSI Database (Pey et al., 2014) (https://portail.betsi.cnrs.fr/)
Araneae	Daily activity period	Diurnal ; nocturnal (1/0)	Bonte et al., 2006; Cardoso et al., 2011; Isaia et al., 2006
	Annual activity period	Spring ; summer ; winter ; autumn (1/0)	araneae.nmbe.ch
	Ballooning (dispersal mode)	1 = Yes ; 0 = No	Birkhofer et al., 2015; Bonte et al., 2006
	Stratum use	Ground ; vegetation (1/0)	Birkhofer et al., 2015; Cardoso et al., 2011
	Hunting strategy	aerial_web ; sheet_web ; cursorial (1/0 for each trait level)	Birkhofer et al., 2015
	Body size (mm)	1.75 – 10.57	BETSI Database (Pey et al., 2014)
Birds	Habitat specialism	0.23-2.39	Julliard et al., 2004
	Breeding activity period	Early (i.e. March/April); Late (i.e. after May)	Cramp et al., 1983
	Hunting strategy	Gleaning ; pursuit ; pouncing ; digging ; grazing ; scavenging (1/0)	Storchová & Hořák, 2018
	Stratum use	Ground ; vegetation ; aerial (1/0)	Storchová & Hořák, 2018
	% of vertebrates in diet (0-100)	0–100	Snow and Perrins, 1998
	% of insect in diet (0-100)	0–0.95	Snow and Perrins, 1998
	% of vegetables in diet (0-100)	0–0.99	Snow and Perrins, 1998
	Number of eggs per year	3-30	Storchová & Hořák, 2018
	Body size (cm)	12.5-71	Storchová & Hořák, 2018

Appendix S1: Details for the calculation of Community Trophic Index (CTI) of birds and Carabidae communities.

CTI is an indicator of mean trophic level. It was calculated based on the proportion of plants (*trophic level = 1*), invertebrates (*trophic level = 2*) and vertebrates (*trophic level = 3*) in the diet of each bird species from Jeliazkov et al., (2016) and on the proportion of detritus (*trophic level = 1*), plants (*trophic level = 2*) and preys (*trophic level = 3*) in the diet of each Carabidae species from BETSI Database (http://betsi.cesab.org). For each species, a trophic index (STI) was computed by summing the three proportions weighted by 1 for the first, 2 for the second and 3 for the third trophic level. Thus, a species at the top of the food chain will have a higher STI than an herbivorous species. The CTI index corresponds to the community mean of STI weighted by each species relative abundance.

Appendix S2: Details for the calculation of standardized estimates

Standardized estimates could not be calculated for glmmPQL class models. Following Grace et al. (2018), we calculated standardized estimates based on observation empirical approach using R^2. For each linear model in the global SEM's model, we extracted R^2 of response variable which is the squared correlation between the raw vs. fitted values. We have then obtained the variance of the raw observations and multiplied them by the ratio of standard deviation of predicted (ÿ) on standard deviation of observed values on y.

Example of code:

```
R2 <- cor(data$y, predict(model, type = "response"))^2 # non-linear
predictions

sd.yhat <- sqrt(var(predict(model, type = "link"))/R2)

coef(model)[2,] * sd(data$x)/sd.yhat
```

More details online https://jslefche.github.io/sem_book/coefficients.html#scaling-to-other-non-normal-distributions

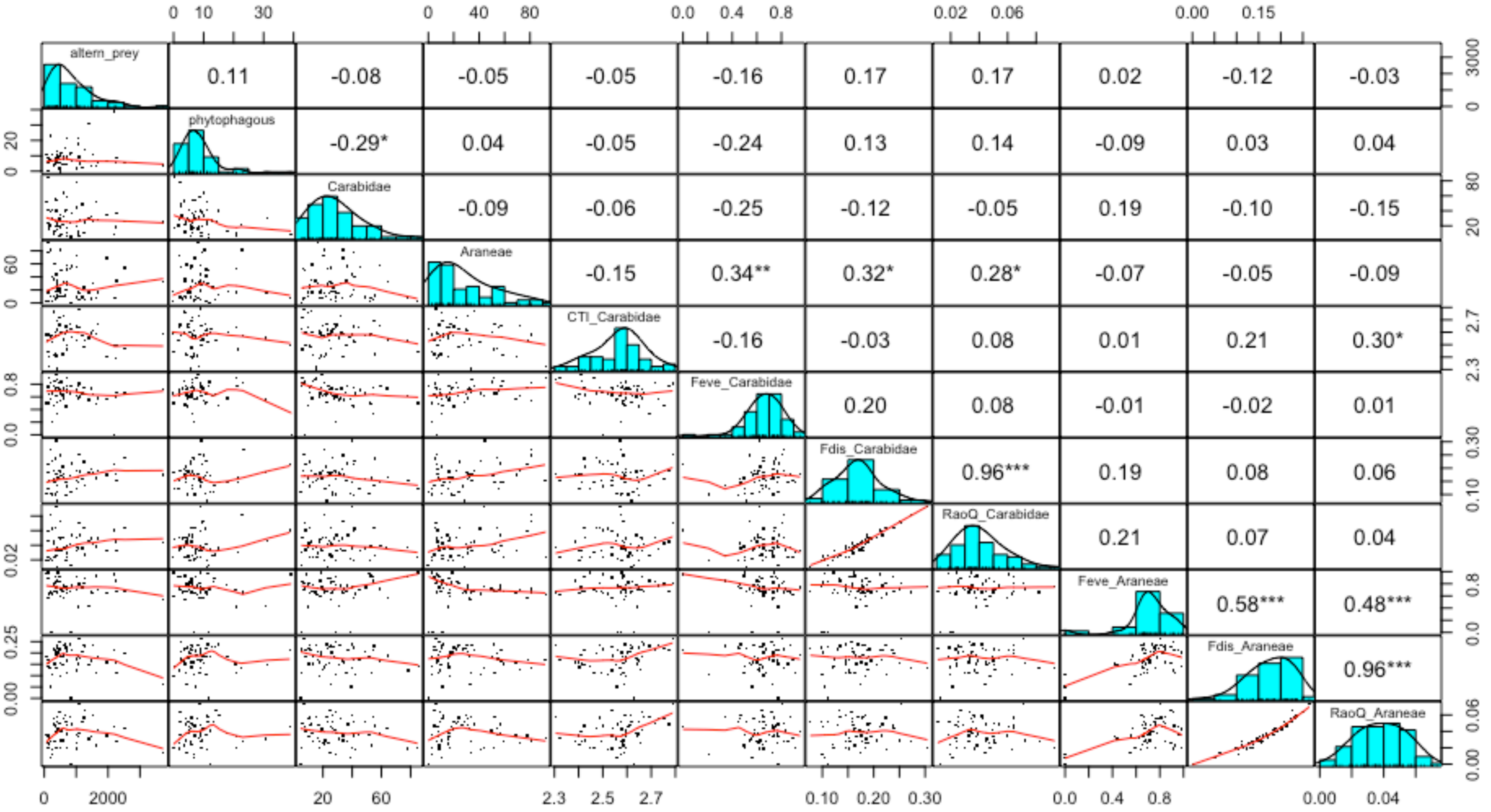

Figure S2: Correlation matrix of species abundances, trophic level and functional variables. The distribution of each of the variables is shown on the diagonal. At the bottom of the diagonal, the scatter plots are shown with the trend curve. On the top of the diagonal, correlation coefficients and significance levels (asterisks) are shown. The library ’psych’ and the function ‘pairs.panels’ have been used.

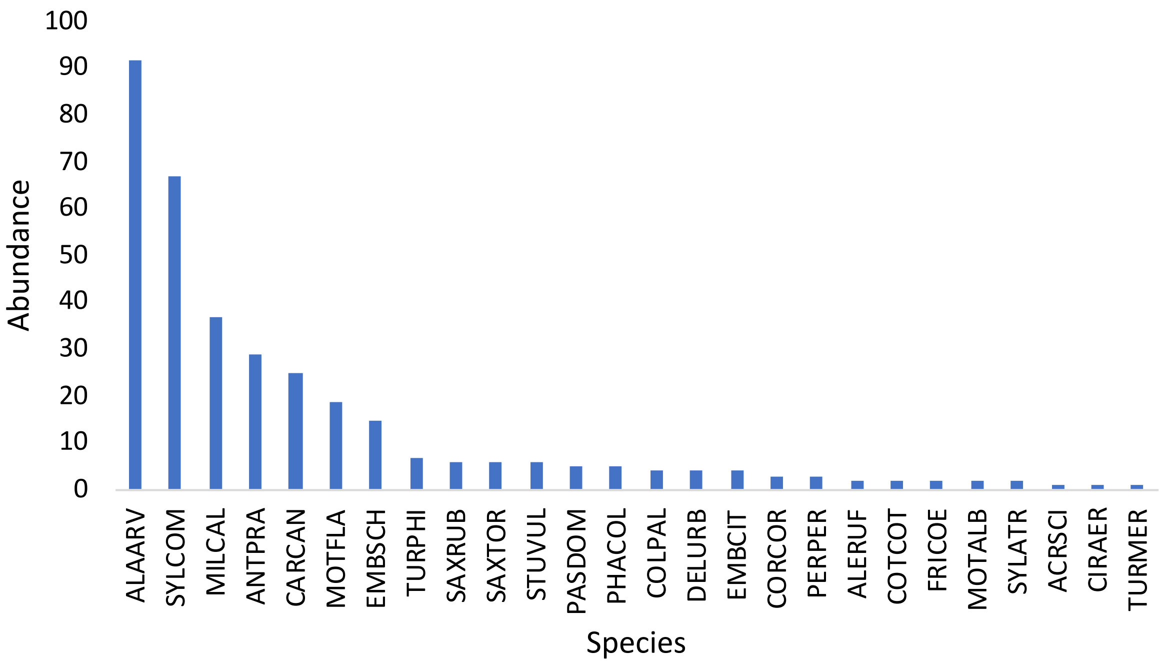

Figure S3: Cumulated abundance of birds' species sampled on control plots.

Species codes (STOC-EPS), ALAARV: *Alauda arvensis (Eurasian skylark)*; SYLCOM: *Sylvia communis (Common whitethroat)*; MILCAL: *Emberiza calandra (Corn bunting)*; ANTPRA: *Anthus pratensis (Meadow pipit)*; CARCAN: *Linaria cannabina (Common linnet)*; MOTFLA: *Motacilla flava (Western yellow wagtail)*; EMBSCH: *Emberiza schoeniclus (Common reed bunting)*; TURPHI: *Turdus philomelos (Song thrush)*; SAXRUB: *Saxicola rubetra (Winchat)*; SAXTOR: *Saxicola rubicola (European stonechat)*; STUVUL: *Sturnus vulgaris (Common starling)*; PASDOM: *Passer domesticus (House sparrow)*; PHACOL: *Phasianus colchicus (Common pheasant)*; COLPAL: *Columba palumbus (Common wood pigeon)*; DELURB: *Delichon urbicum (Common house martin)*; EMBCIT: *Emberiza citrinella (Yellowhammer)*; CORCOR: *Corvus corone (Carrion crow)*; PERPER: *Perdix perdix (Grey partridge)*; ALERUF: *Alectoris rufa (Red-legged partridge)*; COTCOT: *Coturnix coturnix (Common quail)*; FRICOE: *Fringilla coelebs (Common chaffinch)*; MOTALB: *Motacilla alba (White wagtail)*; SYLATR: *Sylvia atricapilla (Eurasian blackcap)*; ACRSCI: *Acrocephalus scirpaceus (Eurasian reed warbler)*; CIRAER: *Circus aeruginosus (Western marsh harrier)*; TURMER: *Turdus merula (Common blackbird)*

Table S4: Effect of the bird exclusion cage and the year on the different response variables (Anova type II). Significant p-values are written in bold. 'Other predators' are the sum of abundances of Staphylinidae, Opiliones and Chilopoda individuals. 'Phytophagous insects' include Thysanoptera, Cicadomorpha, Curculionidae, Nitidulidae and Chrysomelidae.

Response variable (Abundance)	Predictor	Chisq	Df	Pr(>Chisq) Anova	R^2 marginal
Carabidae	Cage	0.18	1	0.67	0.27
	**Year**	7.02	1	**$8.07 \cdot 10^{-3}$ ****	
	Cage x Year	1.02	1	0.31	
Araneae	**Cage**	8.02	1	**$4.63 \cdot 10^{-3}$ ****	0.03
	Year	0.37	1	0.54	
	Cage x Year	0.26	1	0.61	
Others predators	Cage	0.78	1	0.38	0.01
	Year	0.16	1	0.69	
	Cage x Year	0.02	1	0.88	
Alternative preys	Cage	0.87	1	0.35	0.10
	Year	2.87	1	0.090.	
	Cage x Year	0.02	1	0.88	
Phytophagous insects	Cage	0.13	1	0.72	0.00
	Year	0.04	1	0.85	
	Cage x Year	0.19	1	0.67	
CTI Carabidae	Cage	0.76	1	0.38	0.02
	Year	0.33	1	0.56	
	Cage x Year	0.59	1	0.44	
RaoQ Carabidae	Cage	0.46	1	0.50	0.10
	**Year**	4.11	1	**0.04 ***	
	Cage x Year	1.09	1	0.30	
Feve Carabidae	Cage	0.01	1	0.93	0.15
	**Year**	5.14	1	**0.02 ***	
	**Cage x Year**	4.91	1	**0.03 ***	
RaoQ Araneae	Cage	0.50	1	0.48	0.07
	Year	3.18	1	0.07.	
	Cage x Year	0.12	1	0.73	
Feve Araneae	Cage	0.07	1	0.80	0.01
	Year	0.60	1	0.44	
	Cage x Year	0.01	1	0.94	

Appendix S3: Results of the best model for taxonomic (A), trophic level (B) and functional approach (C)

Table S5: Results of model 1 with treatment frequency index as agricultural variable built with the taxonomic approach (A).

**Model 1 Response**	**Predictor**	**Estimates**	**Standard error**	**DF**	**Critical value**	**P-value**	**Standardized estimates**
Carabidae	Birds	-0.0166	0.0084	29	-1.9715	*0.0583*	-0.2678
Carabidae	TFI	-0.0998	0.0481	28	-2.0733	**0.0475***	-0.4124
Araneae	Birds	0.0107	0.0068	27	1.5807	0.1156	0.0976
Araneae	Carabidae	-0.0027	0.0057	27	-0.4774	0.6369	-0.0593
Araneae	Others predators	0.0025	0.0093	27	0.2688	0.7901	0.0299
Araneae	TFI	-0.1094	0.0838	28	-1.3049	0.2026	-0.2544
Others predators	Birds	0.0037	0.0072	28	0.5160	0.6099	0.0578
Others predators	Carabidae	0.0055	0.0040	28	1.3593	0.1849	0.2045
Others predators	TFI	-0.0619	0.0544	28	-1.1382	0.2647	-0.2451
Phytophagous insects	Birds	-0.0077	0.0087	26	-0.8806	0.3866	-0.1030
Phytophagous insects	Carabidae	-0.0061	0.0061	26	-2.2478	**0.0333***	**-0.4432**
Phytophagous insects	Others predators	0.0135	0.0093	26	1.4478	0.1596	0.2376
Phytophagous insects	Araneae	0.0026	0.0042	26	0.6093	0.5476	0.1107
Phytophagous insects	TFI	-0.0197	0.0636	28	-0.3105	0.7585	-0.0677
Alternative preys	Carabidae	-0.0075	0.0039	26	-1.9455	*0.0626*	-0.1744
Alternative preys	Others predators	0.0086	0.0087	27	0.9822	0.3351	0.1081
Alternative preys	Araneae	-0.0096	0.0041	26	-2.3503	**0.0266***	**-0.2964**
Alternative preys	Phytophagous	0.0184	0.0079	26	2.3184	**0.0286***	**0.1672**
Alternative preys	TFI	-0.1354	0.0795	28	-1.7045	0.0994	-0.3324

Table S6: Results of model 2 with tillage as agricultural variable built with the taxonomic approach (A).

**Model 2 Response**	**Predictor**	**Estimates**	**Standard error**	**DF**	**Critical value**	**P-value**	**Standardized estimates**
Carabidae	Birds	-0.0151	0.0084	29	-1.7968	*0.0828*	-0.2407
Carabidae	Tillage	0.0965	0.0557	28	1.7330	*0.0941*	0.3493
Araneae	Birds	0.0112	0.0067	27	1.6611	0.1083	0.1010
Araneae	Carabidae	-0.0012	0.0057	27	-0.2067	0.8378	-0.0254
Araneae	Others predators	0.0037	0.0094	27	0.3921	0.6981	0.0435
Araneae	Tillage	-0.0082	0.0993	28	-0.0824	0.9349	-0.0167
Others predators	Birds	0.0044	0.0073	28	0.6119	0.5455	0.0683
Others predators	Carabidae	0.0053	0.0040	28	1.3377	0.1918	0.1980

Others predators	Tillage	0.0884	0.0598	28	1.4790	0.1503	0.3083
Phytophagous insects	Birds	-0.0077	0.0086	26	-0.8918	0.3807	-0.1014
Phytophagous insects	Carabidae	-0.0120	0.0059	26	-2.0163	*0.0542*	-0.3826
Phytophagous insects	Others predators	0.0159	0.0092	26	1.7239	1.7239	0.2773
Phytophagous insects	Araneae	0.0024	0.0041	26	0.6027	0.5519	0.1039
Phytophagous insects	Tillage	-0.0881	0.0710	28	-1.2416	0.2247	-0.2645
Alternative preys	Carabidae	-0.0072	0.0039	26	-1.8583	*0.0749*	-0.1666
Alternative preys	Others predators	0.0077	0.0088	26	0.8787	0.3876	0.0977
Alternative preys	Araneae	-0.0078	0.0041	26	-1.9322	*0.0643*	-0.2411
Alternative preys	Phytophagous	0.0197	0.0081	26	2.4390	**0.0219***	**0.1791**
Alternative preys	Tillage	0.1455	0.0876	28	1.6609	0.1079	0.3165

Table S7: Results of the best model (model 4 with tillage) among trophic level approach.

Model 4 Response	Predictor	Estimates	Standard error	DF	Critical value	P-value	Standardized estimates
Carabidae	CTI Birds	-0.0258	0.0716	29	-0.3605	0.7211	0.045
CTI Carabidae	Tillage	0.0172	0.0087	28	1.9897	*0.0565*	*0.525*
Araneae	CTI Birds	0.1481	0.0549	29	2.6993	**0.0115***	**0.133**
Phytophagous insects	CTI Birds	-0.0277	0.0744	26	-0.3722	0.7128	-0.037
Phytophagous insects	CTI Carabidae	-1.7400	0.7118	26	-2.4445	**0.0216***	**-0.329**
Phytophagous insects	Araneae	0.0029	0.0041	26	0.7008	0.4897	0.121
Phytophagous insects	Carabidae	-0.0120	0.0056	26	-2.1523	**0.0408***	**-0.382**
Alternative preys	CTI Carabidae	-1.1289	0.6056	27	-1.8640	*0.0732*	*-0.158*
Alternative preys	Araneae	-0.0083	0.0043	27	-1.9411	*0.0628*	*-0.260*
Alternative preys	Carabidae	-0.0088	0.0042	27	-2.1287	**0.0426***	**-0.209**

Table S8: Results of the best model (model 6 for RaoQ index and model 8 for Feve index) among functional approach.

Response model 6	Predictor	Estimates	Standard error	DF	Critical value	P-value	Standardized estimates
Carabidae	RaoQ Birds	0.5242	2.7114	29	0.1933	0.8480	0.025
Araneae	RaoQ Birds	6.6768	2.1734	28	3.0720	**0.0047****	**0.161**
Araneae	RaoQ Carabidae	5.6565	3.5474	28	1.5945	0.1220	0.116
RaoQ Carabidae	Tillage	-0.0003	0.0015	28	-0.2083	0.8365	-0.064
RaoQ Araneae	Tillage	0.0011	0.0013	28	0.7965	0.4325	0.324
Phytophagous insects	RaoQ Carabidae	-5.3718	13.1591	25	-0.4082	0.6866	-0.067
Phytophagous insects	RaoQ Araneae	17.4624	14.5696	25	1.1986	0.2419	0.201
Phytophagous insects	RaoQ Birds	-12.9987	8.2435	25	-1.5768	0.1274	-0.190

Phytophagous insects	Carabidae	-0.0200	0.0152	25	-1.3106	0.2019	-0.259
Phytophagous insects	Araneae	0.0063	0.0112	25	0.5587	0.5813	0.108
Alternative preys	RaoQ Carabidae	1.2021	4.3320	26	0.2775	0.7836	0.028
Alternative preys	RaoQ Araneae	-3.1760	3.5760	26	-0.8882	0.3826	-0.068
Alternative preys	Carabidae	-0.0057	0.0044	26	-1.3091	0.2020	-0.139
Alternative preys	Araneae	-0.0069	0.0044	26	-1.5698	0.1286	-0.223
**Response model 8**	**Predictor**	**Estimates**	**Standard error**	**DF**	**Critical value**	**P-value**	**Standardized estimates**
Carabidae	Feve Birds	-0.1141	0.1304	29	-0.8750	0.3888	-0.112
Araneae	Feve Birds	0.2791	0.0989	28	2.8209	**0.0087****	**0.142**
Araneae	Feve Carabidae	0.5741	0.5233	28	1.0972	0.2819	0.113
Feve Carabidae	Tillage	-0.0003	0.0135	28	-0.0220	0.9826	-0.017
Feve Araneae	Tillage	0.0068	0.0161	28	0.4228	0.6756	0.055
Phytophagous insects	Feve Carabidae	-1.4040	0.3600	25	-3.9001	**0.0006*****	**-0.407**
Phytophagous insects	Feve Araneae	-0.2513	0.3102	25	-0.8102	0.4255	-0.091
Phytophagous insects	Feve Birds	-0.0972	0.1353	25	-0.7188	0.4789	-0.073
Phytophagous insects	Carabidae	-0.0128	0.0056	25	-2.2980	**0.0302***	**-0.403**
Phytophagous insects	Araneae	0.0065	0.0039	25	1.6681	0.1078	0.272
Alternative preys	Feve Carabidae	-0.6570	0.3009	26	-2.1835	**0.0382***	**-0.143**
Alternative preys	Feve Araneae	0.2512	0.2906	26	0.8645	0.3952	0.068
Alternative preys	Carabidae	-0.0093	0.0043	26	-2.1583	**0.0403***	**-0.220**
Alternative preys	Araneae	-0.0068	0.0042	26	-1.6164	0.1181	-0.212

Appendix S4: Results for the functional approach with functional dissimilarity index

Table S9: Results of functional dissimilarity models

Approach	Model	Response	R^2 marginal	R^2 conditional	Fischer's C Test	DF	P-value	AIC
Functional dissimilarity	9 (TFI)	Carabidae	0.00	0.85	38.939	26	0.049	102.939
		Araneae	0.04	0.95				
		Alternative preys	0.06	1.00				
		Phytophagous insects	0.14	0.72				
		Fdis Carabidae	0.14	0.35				
		Fdis Araneae	0.00	0.18				
	10 (Tillage)	Carabidae	0.00	0.85	33.229	26	0.156	97.229
		Araneae	0.04	0.95				
		Alternative preys	0.06	1.00				
		Phytophagous insects	0.14	0.72				
		Fdis Carabidae	0.00	0.35				
		Fdis Araneae	0.00	0.18				

Table S10: Results of the best model (model 10) for the functional dissimilarity index.

**Response Model 10**	**Predictor**	**Estimates**	**Standard error**	**DF**	**Critical value**	**P-value**	**Standardized estimates**
Carabidae	Fdis Birds	0.0021	0.5854	29	0.0035	0.9972	0.000
Araneae	Fdis Birds	1.5031	0.4563	28	3.2944	**0.0027****	**0.166**
Araneae	Fdis Carabidae	2.2730	1.3736	28	1.6548	0.1091	0.126
Fdis Carabidae	Tillage	-0.0006	0.0042	28	-0.1338	0.8945	-0.040
Fdis Araneae	Tillage	0.0020	0.0045	28	0.4414	0.6623	0.202
Phytophagous insects	Fdis Carabidae	-1.6744	1.8675	25	-0.8966	0.3785	-0.140
Phytophagous insects	Fdis Araneae	-0.2912	1.4332	25	-0.2032	0.8406	-0.028
Phytophagous insects	Fdis Birds	-0.8561	0.7476	25	-1.1452	0.2630	-0.144
Phytophagous insects	Carabidae	-0.0112	0.0057	25	-1.9521	*0.0622*	-0.363
Phytophagous insects	Araneae	0.0053	0.0045	25	1.1836	0.2477	0.227
Alternative preys	Fdis Carabidae	-0.3335	1.6959	26	-0.1967	0.8456	-0.021
Alternative preys	Fdis Araneae	-0.6287	1.0778	26	-0.5834	0.5647	-0.045
Alternative preys	Carabidae	-0.0064	0.0044	26	-1.4504	0.1589	-0.154
Alternative preys	Araneae	-0.0068	0.0045	26	-1.5192	0.1408	-0.216

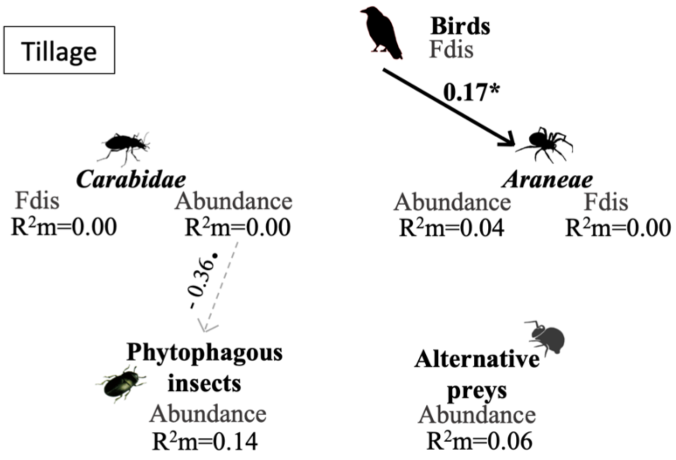

Figure S4: Structural equation modelling showing direct and indirect relationships between the functional dissimilarity (Fdis) of predators and the abundance of phytophagous arthropods (model 10 Table S10). Black and grey lines indicate positive and negative relationships, respectively. Solid lines represent significant relationships ($p<0.05$) and dotted lines represent trends ($0.05<p<0.10$). We reported standardised estimates (with '*' for significant relationships and '.' for trends) for each arrow and marginal R^2 (R^2m). Phytophagous insects include Thysanoptera, Cicadomorpha, Curculionidae, Nitidulidae, and Chrysomelidae. Alternative preys include Collembola and Acari.

References

Baulechner, D., Diekötter, T., Wolters, V., & Jauker, F. (2019). Converting arable land into flowering fields changes functional and phylogenetic community structure in ground beetles. *Biological Conservation*, *231*, 51–58. https://doi.org/10.1016/j.biocon.2019.01.005

Birkhofer, K., Smith, H. G., Weisser, W. W., Wolters, V., & Gossner, M. M. (2015). Land-use effects on the functional distinctness of arthropod communities. *Ecography*, *38*(9), 889–900. https://doi.org/10.1111/ecog.01141

Bonte, D., Lens, L., & Maelfait, J.-P. (2006). Sand dynamics in coastal dune landscapes constrain diversity and life-history characteristics of spiders. *Journal of Applied Ecology*, *43*(4), 735–747. https://doi.org/10.1111/j.1365-2664.2006.01175.x

Brose, U. (2010). Body-mass constraints on foraging behaviour determine population and food-web dynamics. *Functional Ecology*, *24*(1), 28–34. https://doi.org/10.1111/j.1365-2435.2009.01618.x

Cardoso, P., Pekár, S., Jocqué, R., & Coddington, J. A. (2011). Global Patterns of Guild Composition and Functional Diversity of Spiders. *PLoS ONE*, *6*(6). https://doi.org/10.1371/journal.pone.0021710

Clavel, J., Julliard, R., & Devictor, V. (2011). Worldwide decline of specialist species: Toward a global functional homogenization? *Frontiers in Ecology and the Environment*, *9*(4), 222–228. https://doi.org/10.1890/080216

Cole, L. J., McCracken, D. I., Dennis, P., Downie, I. S., Griffin, A. L., Foster, G. N., Murphy, K. J., & Waterhouse, T. (2002). Relationships between agricultural management and ecological groups of ground beetles (Coleoptera: Carabidae) on Scottish farmland. *Agriculture, Ecosystems & Environment*, *93*(1–3), 323–336. https://doi.org/10.1016/S0167-8809(01)00333-4

Cramp, S., Simmons, K. L. E., Brooks, D. C., Collar, N. J., Dunn, E., Gillmor, R., ... & Wilson, M. G. (1983). *Handbook of the birds of Europe, the Middle East and North Africa. The birds of the Western Palearctic*

Forsythe, T. G. (2009). Locomotion in ground beetles (Coleoptera carabidae): An interpretation of leg structure in functional terms. *Journal of Zoology*, *200*(4), 493–507. https://doi.org/10.1111/j.1469-7998.1983.tb02811.x

Gossner, M. M., Simons, N. K., Achtziger, R., Blick, T., Dorow, W. H. O., Dziock, F., Köhler, F., Rabitsch, W., & Weisser, W. W. (2015). A summary of eight traits of Coleoptera, Hemiptera, Orthoptera and Araneae, occurring in grasslands in Germany. *Scientific Data*, *2*, 150013. https://doi.org/10.1038/sdata.2015.13

Grace, J. B., Johnson, D. J., Lefcheck, J. S., & Byrnes, J. E. K. (2018). Quantifying relative importance: Computing standardized effects in models with binary outcomes. *Ecosphere*, *9*(6), e02283. https://doi.org/10.1002/ecs2.2283

Greenop, A., Woodcock, B. A., Wilby, A., Cook, S. M., & Pywell, R. F. (2018). Functional diversity positively affects prey suppression by invertebrate predators: A meta-analysis. *Ecology*, *99*(8), 1771–1782. https://doi.org/10.1002/ecy.2378

Hughes, R. (Ed.). (2009). *Diet selection: an interdisciplinary approach to foraging behaviour*. John Wiley & Sons.

Isaia, M., Bona, F., & Badino, G. (2006). Influence of Landscape Diversity and Agricultural Practices on Spider Assemblage in Italian Vineyards of Langa Astigiana (Northwest Italy). *Environmental Entomology*, *35*, 297–307. https://doi.org/10.1603/0046-225X-35.2.297

Jeliazkov, A., Mimet, A., Chargé, R., Jiguet, F., Devictor, V., & Chiron, F. (2016). Impacts of agricultural intensification on bird communities: New insights from a multi-level and multi-facet approach of biodiversity. *Agriculture, Ecosystems & Environment*, *216*, 9–22. https://doi.org/10.1016/j.agee.2015.09.017

Julliard, R., Jiguet, F., & Couvet, D. (2004). Common birds facing global changes: What makes a species at risk? *Global Change Biology*, *10*(1), 148–154. https://doi.org/10.1111/j.1365-2486.2003.00723.x

Lack, D. (1954). The natural regulation of animal numbers. *The Natural Regulation of Animal Numbers.*

Loeuille, N., Barot, S., Georgelin, E., Kylafis, G., & Lavigne, C. (2013). Chapter Six - Eco-Evolutionary Dynamics of Agricultural Networks: Implications for Sustainable Management. In G. Woodward & D. A. Bohan (Eds.), *Advances in Ecological Research* (Vol. 49, pp. 339–435). Academic Press. https://doi.org/10.1016/B978-0-12-420002-9.00006-8

Martin, E. A., Dainese, M., Clough, Y., Báldi, A., Bommarco, R., Gagic, V., Garratt, M. P. D., Holzschuh, A., Kleijn, D., Kovács-Hostyánszki, A., Marini, L., Potts, S. G., Smith, H. G., Al Hassan, D., Albrecht, M., Andersson, G. K. S., Asís, J. D., Aviron, S., Balzan, M. V., … Steffan-Dewenter, I. (2019). The interplay of landscape composition and configuration: New pathways to manage functional biodiversity and agroecosystem services across Europe. *Ecology Letters*. https://doi.org/10.1111/ele.13265

Naef-Daenzer, L., Naef-Daenzer, B., & Nager, R. G. (2000). Prey selection and foraging performance of breeding Great Tits Parus major in relation to food availability. *Journal of Avian Biology*, *31*(2), 206–214. https://doi.org/10.1034/j.1600-048X.2000.310212.x

Northfield, T. D., Barton, B. T., & Schmitz, O. J. (2017). A spatial theory for emergent multiple predator–prey interactions in food webs. *Ecology and Evolution*, *7*(17), 6935–6948. https://doi.org/10.1002/ece3.3250

Pedley, S. M., & Dolman, P. M. (2014). Multi-taxa trait and functional responses to physical disturbance. *Journal of Animal Ecology*, *83*(6), 1542–1552. https://doi.org/10.1111/1365-2656.12249

Perović, D. J., Gámez-Virués, S., Landis, D. A., Wäckers, F., Gurr, G. M., Wratten, S. D., You, M.-S., & Desneux, N. (2018). Managing biological control services through multi-trophic trait interactions: Review and guidelines for implementation at local and landscape scales. *Biological Reviews*, *93*(1), 306–321. https://doi.org/10.1111/brv.12346

Petersen, L. F., & Woltz, J. M. (2015). Diel variation in the abundance and composition of the predator assemblages feeding on aphid-infested soybean. *BioControl*, *60*(2), 209–219. https://doi.org/10.1007/s10526-014-9631-6

Pey, B., Nahmani, J., Auclerc, A., Capowiez, Y., Cluzeau, D., Cortet, J., Decaëns, T.,

Deharveng, L., Dubs, F., Joimel, S., Briard, C., Grumiaux, F., Laporte, M.-A., Pasquet, A., Pelosi, C., Pernin, C., Ponge, J.-F., Salmon, S., Santorufo, L., & Hedde, M. (2014). Current use of and future needs for soil invertebrate functional traits in community ecology. *Basic and Applied Ecology*, *15*(3), 194–206. https://doi.org/10.1016/j.baae.2014.03.007

Ribera, I., Dec, S. D., Downie, I. S., & Foster, G. N. (2001). *Effect of land disturbance and stress on species traits of ground beetle assemblages*. *82*(4), 26.

Roubinet, E., Birkhofer, K., Malsher, G., Staudacher, K., Ekbom, B., Traugott, M., & Jonsson, M. (2017). Diet of generalist predators reflects effects of cropping period and farming system on extra- and intraguild prey. *Ecological Applications*, *27*(4), 1167–1177. https://doi.org/10.1002/eap.1510

Schmitz, O. J. (2009). Effects of predator functional diversity on grassland ecosystem function. *Ecology*, *90*(9), 2339–2345. https://doi.org/10.1890/08-1919.1

Snow, D. W., Gillmor, R., & Perrins, C. M. (1998). *The birds of the Western Palearctic: Non-passerines*. Oxford University Press.

Storchová, L., & Hořák, D. (2018). Life-history characteristics of European birds. *Global Ecology and Biogeography*, *27*(4), 400–406. https://doi.org/10.1111/geb.12709
